# Effectiveness of High-Flow Nasal Cannula (RAM Cannula) With T-piece Resuscitator to Deliver Continuous Positive Airway Pressure (CPAP) During Neonatal Transport

**DOI:** 10.7759/cureus.61514

**Published:** 2024-06-02

**Authors:** Nilesh Darverkar, Anand Bhutada, Yash Banait, Satish Deopujari, Brij Raj Singh

**Affiliations:** 1 Pediatrics, Datta Meghe Medical College, Datta Meghe Institute of Higher Education and Research, Nagpur, IND; 2 Pediatrics, Nelson Hospital, Nagpur, IND; 3 Pediatrics, Indira Gandhi Medical College, Nagpur, IND; 4 Anatomy, Datta Meghe Medical College, Datta Meghe Institute of Higher Education and Research, Nagpur, IND

**Keywords:** newborns, positive end-expiratory pressure (peep), continuous positive airway pressure (cpap), functional residual capacity (frc), ram’s cannula

## Abstract

Background

Newborns frequently experience respiratory distress (RD), necessitating preventive management during transportation. The use of Continuous Positive Airway Pressure (CPAP) is crucial in mitigating RD in neonates, particularly during transit. This study aims to assess the feasibility and efficacy of utilizing a RAM cannula (Neotech Products, Valencia, USA) with a T-piece resuscitator to deliver CPAP during neonatal transport. The objective is to evaluate the response of transported neonates to this intervention, including improvements in distress, surfactant requirements, ventilator dependency, and complications.

Method and material

Neonates with RD qualifying for CPAP support at birth and requiring transport to Neonatal Intensive Care Unit (NICU) care were included. The average duration of transport was 38 minutes (range 12 minutes to 2 hours). RAM cannula with a T-piece resuscitator was used for CPAP delivery during transportation. Vital parameters and interventions were monitored during transit, and outcomes were compared with inborn neonates receiving standard CPAP in the labor room.

Results

Out of 48 babies, nine babies required surfactant, and four needed invasive ventilation, with three developing a nasal injury. Compared to in-house preterm babies, these babies had more Positive End Expiratory Pressure (PEEP) knob adjustment, desaturation episodes, late surfactant administration, and intubation needs.

Conclusion

A high-flow nasal cannula combined with a T-piece resuscitator emerges as a promising modality for CPAP delivery during neonatal transportation, demonstrating efficacy with minimal complications.

## Introduction

Newborns often experience respiratory distress (RD) stemming from various aetiologies, necessitating meticulous care during transportation [1]. The mere oxygen supplementation is inadequate in such cases, as the delicate pulmonary structures of newborns make them susceptible to deterioration, particularly during transit, where the disease process may precipitate the collapse of smaller alveoli into larger ones. Neonates have inherently low functional residual capacity (FRC), further compromised by pathological progression, thus precipitating RD and subsequent clinical decline [2]. The established literature underscores the efficacy of positive end-expiratory pressure (PEEP) in improving this condition by enhancing FRC, facilitating lung recruitment, and preventing alveolar collapse. In particular, in preterm newborns, early application of PEEP, either in the delivery room or during transport, has been shown to reduce the dependence on surfactants and mechanical ventilation [3]. The success of continuous positive airway pressure (CPAP) is significantly on prompt initiation, effectively mitigating atelectasis, preserving surfactant levels, and curtailing the inflammatory response cascade [4].

However, the practical implementation of PEEP strategies poses challenges, particularly during neonatal transport. Traditional methods such as bubble CPAP or T-piece resuscitators prove cumbersome in a dynamic environment like an ambulance. Currently, there is a dearth of suitable interfaces tailored for the delivery of PEEP to spontaneously breathing newborns and young infants [5]. Variable-flow CPAP remains underutilized in clinical practice. To address this gap, our study explores the feasibility and efficacy of using RAM cannula (Neotech Products, Valencia, USA), commonly associated with high-flow nasal cannula (HFNC) therapy, as an alternative interface during neonatal transportation. Careful selection of the size of the cannula ensures a snug fit within the neonatal nostril, while adherence to established protocols governs the remaining settings [6].

Throughout the study, we scrutinized the response of transported newborns to this novel intervention, monitoring improvement in distress, instances of deterioration, surfactant requirements in premature infants, ventilator dependency, and potential complications. In particular, we excluded cases of RD manifesting after the first 12 hours of life, acknowledging the divergent aetiologies and minimizing confounding variables associated with delayed CPAP application in neonatal outcomes. The objective of the study is to evaluate the effectiveness of the RAM cannula with a T-piece resuscitator in delivering CPAP during neonatal transport.

## Materials and methods

Study design

This observational study evaluated the effectiveness of a RAM cannula with a T-piece resuscitator in delivering CPAP during neonatal transport among residents of Nagpur City, Maharashtra over 6 months from February to August 2023. Ethical approval for the study has been obtained from the Institutional Ethical Committee of Shalinitai Meghe Hospital and Research Centre (SMHRC/IEC/2023/04-78).

Participants

Neonates with RD who qualified for CPAP support (Silverman Andersen respiratory severity score of 6 or more) at birth and required transportation to the Neonatal Intensive Care Unit (NICU) were included. Exclusion criteria comprised newborns with significant co-morbidity like congenital heart disease, age more than 12 hours at the time of transport, and extremely low birth weight newborns.

Transportation equipment and procedure

During transportation, a RAM cannula with a T-piece resuscitator was used, along with a blended oxygen source provided by the Drager incubator (Dräger, Lübeck, Germany) with MVP-10 transport ventilator (Bio-Med Devices, Guilford, USA). Fraction of inspired oxygen (FiO2) levels were adjusted to maintain peripheral oxygen saturation (SpO2) between 90% and 95%. To ensure comfort and safety, the size of the nasal cannula was carefully selected to fit snugly without inducing nasal pressure. Additionally, a padded dressing (duoDERM, Convatec, Reading, United Kingdom) was applied to protect the nasal columella, with care paid to maintain a small space between the columella and the nasal prongs. The FiO2-adjusted flow was maintained at 8 litres/min, while the T-piece resuscitator (Neopuff, Fisher & Paykel Healthcare, Auckland, New Zealand) was securely attached. Before patient connection, the patient end was occluded and positive end-expiratory pressure (PEEP) was adjusted to 5. Subsequently, the T-piece resuscitator was connected to the RAM cannula before attachment to the patient. Larger RAM cannulas were preferred to prevent premature PEEP readings. For very small babies, a mask-covered T-piece was utilized. Throughout the transportation process, patient vitals, including PEEP readings, were diligently monitored to ensure optimal care and safety.

Inclusion criteria

All newborns with respiratory distress who qualify for CPAP support (Silverman Andersen respiratory severity score (SAS) of four or more at birth and need to be transported for Neonatal Intensive Care Unit (NICU)) care were included. The study was conducted in Nagpur city in Maharashtra over a period of six months from February to August 2023. These babies were compared with in-house babies (inborn babies born in the hospital with the availability of a standard bubble CPAP machine in the delivery room). In-house (inborn) babies are also selected for CPAP support using standard indications (SAS score of four or more). As in-house (inborn) babies received CPAP under standard conditions, they are considered the reference for comparison. 

Exclusion criteria

The exclusion criteria were: all newborns with significant comorbidity like congenital heart disease; age more than 12 hours at the time of transport; extremely low birth weight newborns requiring a very small size of RAM cannula.

Statistical analysis

Data entry was done in a Microsoft Excel sheet (Microsoft Corporation, Redmond, USA) and analysis was done using SPSS version 24 (IBM Corp., Armonk, USA). Descriptive statistics (such as frequency, percentages, mean, and standard deviation) were calculated for study variables. Also, for comparisons between groups, chi-square tests were applied to categorical data, at a p-value of < 0.05.

## Results

During the study period of 6 months, a total of 48 newborns were transported using a RAM cannula and T-piece resuscitator to deliver CPAP. The average age of the babies was 6 hours (Range 30 minutes to 12 hours). The average transport time was 38 minutes (range 12 minutes to 2 hours). Table 1 shows the distribution of the transport group and in-house (inborn) group, detailing the number and percentage of babies by gender in each category of gestational age. Especially infants aged 31 to 34 weeks are the largest group, with a balanced gender distribution in the age group.

**Table 1:** Characteristics of patients n - Number; % - Percentage

Gestational Age	Total in-house		Total transport		Male in-house		Male transport		Female in-house		Female transport	
	n	%	n	%	n	%	n	%	n	%	n	%
28 to 30 weeks	30	25%	14	29%	13	11%	6	13%	17	14%	8	17%
31 to 34 weeks	64	52%	24	50%	30	25%	10	21%	34	28%	14	29%
35 to 37 weeks	18	15%	6	13%	10	8%	4	8%	8	7%	2	4%
More than 37 weeks	10	8%	4	8%	6	5%	3	6%	4	3%	1	2%
Total	122	100%	48	100%	59	48%	23	48%	63	52%	25	52%

Table 2 shows interventions during infant transportation in comparison to in-house (inborn) babies, with significant differences highlighted by chi-square analysis (p-value=0.001). Adjustments such as nasal suction, PEEP adjustment, and FiO2 increases show significant differences and emphasize the importance of personalized interventions during infant transportation to effectively meet important needs.

**Table 2:** Comparison between babies with inborn babies who required CPAP since birth n - Number; % - Percentage; PEEP - Positive end-expiratory pressure; FiO2 - Fraction of inspired oxygen

Intervention required	Transport Group		In house patients		Significance level	
	n	%	n	%	Chi-square	p-value
Nasal suction	2	4.10%	6	4.90%	23.173	0.001
Stimulation	2	4.10%	8	6.50%		
Repositioning of canula	3	6.20%	10	8.10%		
Readjustment of the PEEP knob	9	18.70%	0	0%		
Increase in PEEP	5	10.40%	11	9%		
Increase in flow	9	18.70%	18	14.70%		
Desaturation requiring an increase in FiO2	8	16.60%	12	9.80%		
Intubation during transport	3	6.20%	0	0%		

Table 3 shows the complications among patients in the transport group and in-house (inborn) group. As indicated by the chi-square analysis, the p-value (0.930) indicates the need for a surfactant, and the chi-square (0.144) indicating intubation and nasal injury, is not significant, indicating consistent complication rates across the configurations.

**Table 3:** Development of complications n- Number; %-Percentage

Complications	Transport Group		In-house patients		Significance level	
	n	%	n	%	Chi-square	p-value
Need of surfactant	9	18.7%	18	14.7%	0.144	0.930
Intubation	4	8.3%	8	6.5%		
Nasal injury	3	6.2%	8	6.5%		

Nine babies required surfactant and four of them required invasive ventilation a few hours after reaching the hospital. Only three babies developed nasal injuries. These babies were compared to in-house preterm babies, requiring CPAP in the labor room (standard bubble CPAP with Fisher and Paykel machine). The benefits and complications in these babies were compared. It was found that the need for PEEP knob adjustment due to fluctuation in PEEP readings (during transportation, movement of the baby increases, which causes leakage of airflow around nostrils, causing fluctuations in PEEP reading), episodes of desaturation, late surfactant administration, and need for intubation was more in the study group. We need a detailed case-controlled study to evaluate the difference between these babies and inborn babies who do not require any transport. In our study, we did not find any significant difference between these babies and their inborn counterparts at first glance.

## Discussion

The escalating incidence of preterm deliveries concomitant with the growing demand for CPAP underscores the pivotal role of uninterrupted respiratory support in improving neonatal outcomes [7]. Transporting neonates without the provision of PEEP, even for brief durations, poses a risk of lung compromise, emphasizing the criticality of integrating CPAP into neonatal transport protocols. This necessitates a comprehensive approach to meet the burgeoning demand for nasal C-PAP, ensuring safe and effective respiratory support during ambulance transfers overseen by seasoned healthcare professionals.

The efficacy of nasal CPAP in ensuring respiratory stability during land-based ambulance transfers has been validated, particularly when administered by skilled personnel following stringent patient selection criteria and fostering seamless communication between referring and receiving teams [8]. Furthermore, studies have demonstrated that early use of CPAP in premature neonates can reduce the need for surfactants and ventilation [9]. However, the optimal strategies for CPAP titration during neonatal transport remain an area of ongoing research [10,11].

Nonetheless, the current landscape underscores the imperative for technological advancements to optimize CPAP delivery during neonatal transport. A pressing need exists for innovations encompassing airflow generators, FiO2 titration devices, and refined interfaces tailored to meet the unique respiratory needs of neonates in transit [12]. Additionally, emerging studies suggest the potential benefits of integrating CPAP with high-flow nasal cannula therapy for select neonates [13].

Pending the development of dedicated transport-oriented CPAP solutions, it is incumbent upon healthcare providers to navigate these challenges adeptly, leveraging the expertise of specialized transport teams and exhaustively exploring available resources to escalate respiratory support if warranted [14,15]. Notably, our observations reveal that while the lack of warmed and humidified gases during transport presents a notable challenge, our short-term data indicate no discernible adverse effects attributable to this constraint. Nonetheless, addressing this issue remains imperative for enhancing the safety and comfort of neonatal transport in the long term.

Limitation

A constraint of the research is its observational design, which hinders the establishment of causal relationships between the intervention (application of T-piece resuscitation with RAM cannula for CPAP administration) and the results. Furthermore, the single-center methodology and relatively small sample size of the study may limit the applicability of the findings to more diverse neonatal transport settings. Lastly, the results may only apply to these particular populations due to the exclusion of very low birth weight newborns and neonates with substantial comorbidities.

## Conclusions

A high-flow nasal cannula combined with a T-piece resuscitator represents a viable option for administering CPAP to newborns during transportation. This approach demonstrates significant efficacy and a low incidence of complications. However, further evaluation through a comprehensive case-control study is warranted to elucidate its potential benefits and ascertain its safety profile conclusively. Such research endeavors are essential for refining clinical practices and optimizing respiratory support strategies for neonatal transport.
